# Perspective on the phase diagram of cuprate high-temperature superconductors

**DOI:** 10.1038/ncomms11413

**Published:** 2016-05-06

**Authors:** Damian Rybicki, Michael Jurkutat, Steven Reichardt, Czesław Kapusta, Jürgen Haase

**Affiliations:** 1 Institute of Experimental Physics II, University of Leipzig, Faculty of Physics and Earth Sciences, Linnéstrasse 5, Leipzig 04103, Germany; 2 AGH University of Science and Technology, Faculty of Physics and Applied Computer Science, Department of Solid State Physics, al. A. Mickiewicza 30, Krakow 30-059, Poland

## Abstract

Universal scaling laws can guide the understanding of new phenomena, and for cuprate high-temperature superconductivity the influential Uemura relation showed, early on, that the maximum critical temperature of superconductivity correlates with the density of the superfluid measured at low temperatures. Here we show that the charge content of the bonding orbitals of copper and oxygen in the ubiquitous CuO_2_ plane, measured with nuclear magnetic resonance, reproduces this scaling. The charge transfer of the nominal copper hole to planar oxygen sets the maximum critical temperature. A three-dimensional phase diagram in terms of the charge content at copper as well as oxygen is introduced, which has the different cuprate families sorted with respect to their maximum critical temperature. We suggest that the critical temperature could be raised substantially if one were able to synthesize materials that lead to an increased planar oxygen hole content at the expense of that of planar copper.

## Abstract (web summary)

Cuprate superconductors show critical temperatures over 100 K, below which current flows without resistance. Here, the authors show how this temperature is set by material chemistry, leading to a reinterpretation of the cuprate phase diagram and suggestions of how to raise this temperature in the future.

## Graphical abstract

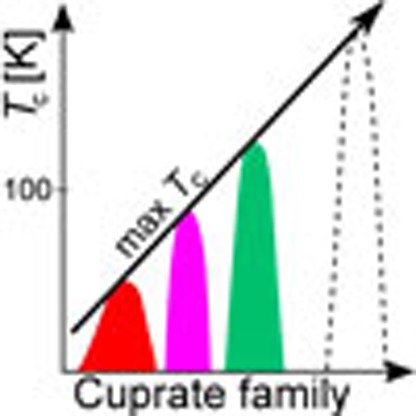

The understanding of the complex properties of the cuprates, and what causes their high critical temperature of superconductivity (*T*_c_), is one of the greatest challenges in condensed matter physics. From it one expects clues that make the synthesis of cuprates with much higher *T*_c_ possible or of how to improve other fundamental properties. In particular, it is still not well understood what sets the very different, maximum *T*_c_'s for different families of materials. An early experimental observation in this regard is the famous Uemura plot^1^. It shows that the maximum *T*_c_ is correlated with the muon spin relaxation rate *σ*_0_ (extrapolated to *T*=0 K) that is proportional to the superfluid density divided by the effective mass (*σ*_0_∝*n*_s_/m*). This relation holds for the underdoped materials and orders different cuprate families with respect to their maximum *T*_c_. The Uemura relation and subsequent scaling laws have remained stimulating, up to now, and some were shown to be valid for other superconductors as well^2^,^3^,^4^,^5^,^6^,^7^,^8^,^9^. While there are various attempts at a theoretical explanation of the Uemura relation^4^,^10^,^11^,^12^, a connection to other experimentally probed properties of cuprates is still lacking, in particular to material chemistry parameters.

The various cuprate families have in common, see Fig. 1a, a CuO_2_ plane and charge reservoir (CR) layers that separate the planes from each other. While the nearly square CuO_2_ plane, defined by the Cu 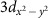 orbital bonding to four O 2*p*_*σ*_ orbitals, is very similar for all systems, the CR chemistry can vary significantly. The nominal hole at Cu (3*d*^9^ configuration) is responsible for strong magnetic correlations that make parent materials antiferromagnetic. Holes or electrons can be added to the CuO_2_ plane by alteration of CR layers. As a result, static magnetism vanishes and new electronic phenomena emerge and the systems become conducting or superconducting. There are many similarities between different cuprate families, and one typically differentiates only between the hole and electron-doped phase diagrams, depicted in Fig. 1b, that appear to show a distinct asymmetry. However, the extent in temperature and doping of the different phases and observed phenomena varies substantially between different families, also for the much more thoroughly investigated hole doped materials. In addition, the comparability between different families is somewhat obstructed with regard to doping. For example, while for La_2−*x*_Sr_*x*_CuO_4_ the doping level can be varied over a large range quite reliably by stoichiometry, interstitial doping with oxygen (O_*δ*_) as, for example, in HgBa_2_CuO_4+*δ*_ leaves uncertainties with regard to the actual doping level. In addition, ionic migration may cause phase separation or other ordering phenomena as in the YBa_2_Cu_3_O_6+*y*_ systems. Given the very different *T*_c_ for various cuprate families, it was questioned whether the average doping level of the CuO_2_ plane is the appropriate chemical parameter for discussing all aspects of the complex physical properties of the cuprates, or whether other parameters should be considered as well, for example, distances within the plane, buckling, disorder, the role of the apical oxygen, or interlayer coupling. Nevertheless, from the understanding of the electronic properties we expect, in particular, clues as how to raise *T*_c_.

Nuclear magnetic resonance (NMR), as a local probe of the magnetic spin susceptibility, focusses mostly on measurements of shift and relaxation caused by the interaction of the nuclear magnetic dipole moment with electronic magnetic moments. However, the nuclear electric quadrupole moment for nuclei with spin *I*>1/2 (*I*=3/2 for ^63,65^Cu, *I*=5/2 for ^17^O) interacts with the local electric field gradient (EFG) causing a quadrupole splitting (*ν*_Q_) of the NMR lines in high magnetic fields. The EFG at the nuclear site is very sensitive to the local charge symmetry, and has been useful for assigning NMR signals to the various lattice positions or detecting inhomogeneous charge distributions in the CuO_2_ plane^13^,^14^. Since the quadrupole splittings of planar Cu and O depend on doping, models have been put forward that attempted to understand these changes in terms of the hole content of certain orbitals, see for example, refs 15, 16, 17, 18 and works cited therein. While trends relating the local charge distribution with *T*_c_ could be established, the uncertainty with regard to the EFG contributions from a variable CR chemistry, limited quantitative agreement with charge contents expected from stoichiometry, as well as insufficient experimental data hampered advances of such analyses.

Here we show that if we plot *T*_c_ versus the planar ^17^O NMR quadrupole splitting, a functional dependence very similar to that of the Uemura plot emerges. This documents that the superfluid density is a function of the EFG at planar O. Based on very recent progress in the understanding of NMR quadrupole splittings in terms of the charge distribution in the CuO_2_ plane^19^ we show that the maximum *T*_c_ increases with the hole content of the planar O 2*p*_*σ*_ orbital, at the expense of that at Cu 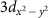. Thus, we identify material chemistry parameters, the hole contents at planar Cu and O, that are largely temperature independent, yet determine the superfluid density at low temperatures. This finding stimulates the use of these orbital hole contents, calculated from NMR literature data^16^,^18^,^19^,^20^,^21^,^22^,^23^,^24^,^25^,^26^,^27^,^28^,^29^,^30^,^31^,^32^,^33^,^34^,^35^,^36^,^37^,^38^,^39^,^40^,^41^,^42^,^43^,^44^,^45^,^46^,^47^,^48^,^49^, to draw a three-dimensional cuprate phase diagram that encompasses all cuprate families and has the superconducting domes ordered according to the maximum *T*_c_. We argue that such a phase diagram might be very useful in discussing the complex properties of the cuprates.

## Results

### Superfluid density and charge densities in the CuO_2_ plane

**Figure 1:**
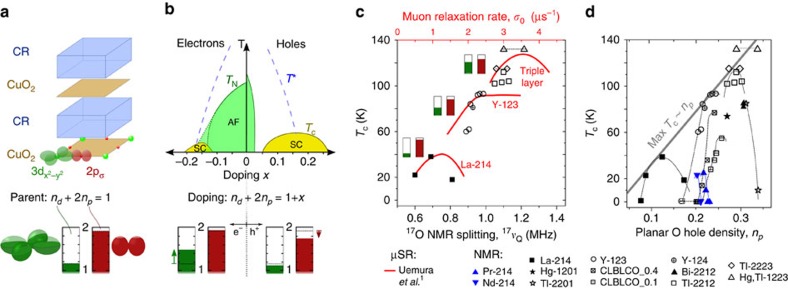
General features of the cuprates. (**a**) The cuprates′ layered structure consists of CR layers and CuO_2_ planes with the bonding orbitals Cu 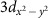 and O 2*p*_*σ*_, which share the nominal 3*d* hole of the Cu^2+^ ion. Columns indicate occupation of Cu 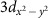 (2−*n*_*d*_) and O 2*p*_*σ*_ (2−*n*_*p*_), with hole contents *n*_*d*_ and *n*_*p*_ measurable with NMR. (**b**) Schematic representation of electronic phase diagram of the cuprates for electron and hole doping *x*: AF phase below Néel temperature (*T*_N_), SC below critical temperature (*T*_c_), and pseudogap regime below pseudogap temperature (T*). Doped electrons (e^−^) go to the Cu 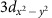 orbital almost exclusively, while doped holes (h^+^) predominantly go to the O 2*p*_*σ*_ orbital, arrows next to columns indicate changes of *n*_*d*_ and *n*_*p*_ caused by doping. (**c**) Solid red: Uemura plot^1^, that is, *T*_c_ versus muon spin relaxation rate (*σ*_0_, upper abscissa); black symbols: *T*_c_ versus planar oxygen quadrupole splitting ^17^*ν*_Q_ (lower abscissa). For list of abbreviations see Table 1. For triple layer Tl-2223 and Hg,Tl-1223 the pairs connected with a dotted line belong to the same sample and correspond to planar O sites of inner and outer layer (smaller splitting corresponds to underdoped inner CuO_2_ layer). (**d**) *T*_c_ versus planar O hole density *n*_*p*_ calculated from ^17^*ν*_Q_ for all available data (see text). Black dotted lines are guides to the eye and connect different doping levels for one family. Solid grey line indicates increase of the maximum *T*_c_ (that is, for the optimal doping level) as a function of *n*_*p*_. AF, antiferromagnetic; SC, superconducting phase.

We plot in Fig. 1c, together with the original Uemura plot (in red), *T*_c_ versus ^17^*ν*_Q_ for similar materials and doping, and find a striking correspondence. This shows that the muon spin relaxation rate deep inside the superconducting state must be tied to the almost temperature independent EFG at the planar O nucleus, which determines the ^17^O NMR splitting measured far above *T*_c_, a rather unanticipated result.

It was confirmed recently, based on NMR data on the electron-doped and parent compounds, that NMR quadrupole splittings provide a quantitative measure of the charge distribution in the CuO_2_ plane of apparently all cuprates^19^. A list of materials with abbreviations is given in Table 1. It was shown that the hole densities in the Cu 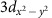 orbital (*n*_*d*_) and the O 2*p*_*σ*_ orbital (*n*_*p*_) in the CuO_2_ plane are related to the experimentally measured splittings ^63^*ν*_Q_ at ^63^Cu and ^17^*ν*_Q_ at ^17^O as follows^18^,^19^:


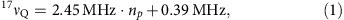



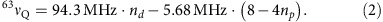


The planar oxygen splitting in equation (1) is only dependent on the hole content *n*_*p*_ of the onsite bonding orbital 2*p*_*σ*_ with the prefactor 2.45 MHz derived from the electric hyperfine interaction experimentally determined with atomic spectroscopy of the O 2*p*^5^ state^18^. The term of 0.39 MHz is due to the charge symmetry at planar oxygen in the ubiquitous CuO_2_ plane, and this term is found to be rather independent on doping and similar for all families^19^. Therefore, one can easily convert the experimentally measured ^17^*ν*_Q_ into a reliable hole content *n*_*p*_. The situation is somewhat more complicated for the Cu splitting in equation (2), which depends on the hole densities of both bonding orbitals. The first term is from the onsite hole content *n*_*d*_ of 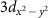 with the prefactor 94.3 MHz, again derived from atomic spectroscopy of Cu 3*d*^9^ state^18^. The second term in equation (2) accounts for the EFG at the Cu nucleus caused by the charge in the bonding orbitals of the four surrounding planar O atoms, with the prefactor derived from the orbital overlap of O 2*p*_*σ*_ with the empty Cu 4*p* and the electric hyperfine interaction of the latter^18^.

First, we use equation (1) and convert all the planar oxygen splittings from the literature to *n*_*p*_. The result is plotted in Fig. 1d, that is, we plot *T*_c_ versus *n*_*p*_ for the different materials. Of course, this plot is very similar to Fig. 1c, but it includes non-superconducting underdoped and parent materials with *T*_c_=0 K. We see that different cuprate families have rather different *n*_*p*_, which results in the sorting of the families as in the Uemura plot. We also recognize that a large *n*_*p*_ is a prerequisite for a high maximum *T*_c_, that is, at optimal doping. In Fig. 1d one can also notice a parabolic-like dependence of *T*_c_ on the oxygen charge *n*_*p*_, which resembles the typical phase diagram that shows a dome-like dependence of *T*_c_ on the average doping level. The correlation between *σ*_0_ and ^17^*ν*_Q_ is lost in the overdoped regime where *σ*_0_ decreases with increasing doping^50^,^51^, which was attributed to a decrease of *n*_*s*_ (ref. 52).

In Fig. 1d, we also included recent results for the electron-doped materials^19^. For Nd_1.85_Ce_0.15_CuO_4_ the superfluid density was reported to be very similar to that of hole doped YBa_2_Cu_3_O_6+*y*_, albeit measured optically and not by muon spin relaxation^3^,^53^,^54^. We find that these results are also in agreement with ^17^*ν*_Q_ splittings (see Supplementary Fig. 1) and corresponding hole contents for those two families, cf. Fig. 1d. Electron doping appears to be less efficient in providing a high *T*_c_, but the rather high oxygen hole contents of the parent materials Pr_2_CuO_4_ and Nd_2_CuO_4_ suggest that hole doping should result in much higher *T*_c_. The so-called infinite layer cuprate Sr_1−*x*_La_*x*_CuO_2_, for which there are no reports of ^17^O splittings, has the highest *T*_c_ among electron-doped families and a very high muon spin relaxation rate (*σ*_0_≈4.5 μs^−1^) (ref. 53), and we expect a high *n*_*p*_. Indeed, a rather high *T*_c_ of more than 100 K was reported in the infinite layer system upon hole doping^55^,^56^.

Clearly, a large *n*_*p*_ is a prerequisite for a high *T*_c_, but is not sufficient, as expected for such a material chemistry parameter. If this empirical relation (max *T*_c_∝*n*_*p*_) remains valid for higher oxygen hole content, the *T*_c_ of the cuprates might be raised substantially by the proper chemistry (we estimate 300–400 K per oxygen hole from the straight line in Fig. 1d).

The splittings of the ^63^Cu NMR lines can only be converted into *n*_*d*_ if there are also ^17^O NMR data available, cf. equation (2). However, there are much less ^17^O splittings reported since the materials have to be enriched with ^17^O (the naturally abundant ^16^O nucleus has spin *I*=0) and therefore only part of ^63^Cu splittings can be converted. (In Supplementary Fig. 1, we plot *T*_c_ versus experimentally measured splittings).

Note that the simple analysis using equations (1) and (2) gives hole densities that are in astonishingly good quantitative agreement with the total charge in the CuO_2_ plane expected from the stoichiometry of the materials^19^, that is,


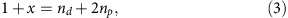


where the factor of 2 accounts for the two O atoms per CuO_2_. This means that the sum of the hole contents *n*_*d*_ and *n*_*p*_ as determined with NMR (r.h.s.) equals the inherent Cu 3*d*^9^ hole content plus the hole content added by doping *x* (l.h.s.). This agreement was shown to apply for electron, as well as hole doping, and different parent materials differ only in terms of the charge transfer between Cu and O^19^. One can therefore also infer from Fig. 1d that compounds with the highest maximum *T*_c_ favour a smaller Cu hole content, and we conclude that it is the transfer of hole density to the O sites that is important for the highest *T*_c_. With this result, one may ask if other properties of the cuprates should be discussed in terms of *n*_*p*_ and *n*_*d*_, as well? This leads us to propose a cuprate phase diagram based on NMR.

### Phase diagram of the cuprates based on NMR

**Figure 2:**
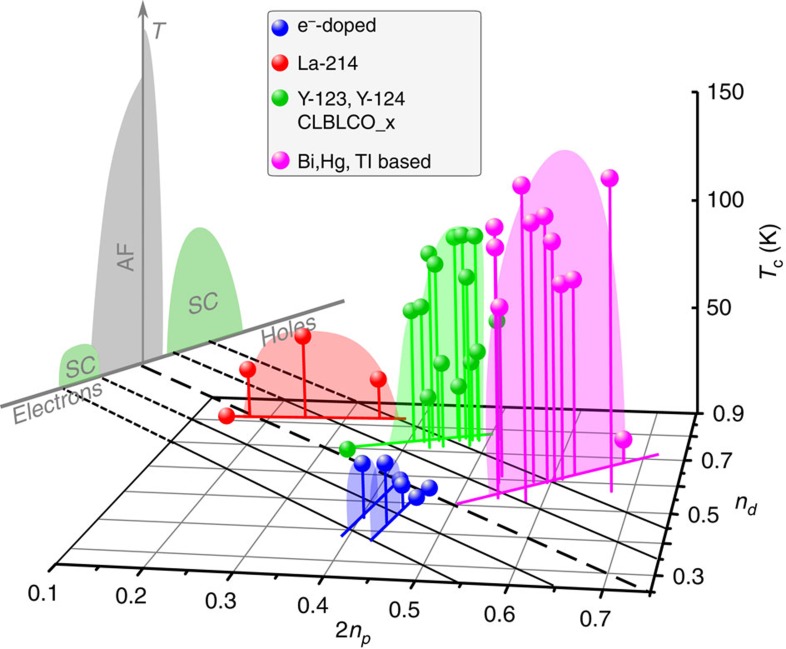
Cuprate phase diagram from NMR. *T*_c_ as a function of oxygen (2*n*_*p*_) and copper (*n*_*d*_) hole content for hole doped La-214, Y-123, Y-124, CLBLCO_x and Bi-, Hg-, Tl-based compounds, as well as electron (e^−^) doped Pr-214 and Nd-214. For list of abbreviations see Table 1. The parent line (dashed bold black) indicates expectation for the undoped case (*n*_*d*_+2*n*_*p*_=1 from *x*=0), parallel lines (thin black) correspond to expectation for doping *x*=*n*_*d*_+2*n*_*p*_−1 changing with a step of 0.1. The commonly used phase diagram (*T* versus *x*) appears as a projection (upper left).

In Fig. 2 we plot *T*_c_ as a function of *n*_*d*_ and 2*n*_*p*_ for all cuprates for which we could find both, Cu and O quadrupole splittings in the literature (see Supplementary Tables 1 and 2) with *n*_*d*_ and *n*_*p*_ calculated from equations (1) and (2). All materials appear in four separate groups, marked by colour: (1) La_2−*x*_Sr_*x*_CuO_4_; (2) YBa_2_Cu_3_O_6+*y*_, and other cuprates of that structure, for example, (Ca_*x*_La_1−*x*_)(Ba_1.75−*x*_La_0.25+*x*_)Cu_3_O_6+*y*_ as well as YBa_2_Cu_4_O_8_; (3) Bi, Tl and Hg based families; and finally, (4) the two electron-doped systems Pr_2−*x*_Ce_*x*_CuO_4_ and Nd_2−*x*_Ce_*x*_CuO_4_. The parent line, that is, the line that is given by *n*_*d*_+2*n*_*p*_=1 (bold dashed line) separates hole doped and electron-doped systems. Note that the lines parallel to the parent line are given by *n*_*d*_+2*n*_*p*_=1+*x*, and represent constant hole (*x*=+0.1, +0.2) or electron (*x*=−0.1, −0.2) doping. While there may be material-specific uncertainties, for example, La_2_CuO_4_ is not located exactly on the parent line, our straightforward analysis uncovers simple systematic trends concerning all cuprates, and we discuss some salient features now.

While *n*_*d*_ and *n*_*p*_ change significantly between different parent compounds along the line *n*_*d*_+2*n*_*p*_=1, antiferromagnetism persists as long as there is one hole per CuO_2_. Such a large range of variation in the charge transfer in different parent compounds, with 2*n*_*p*_ ranging from 0.15 to 0.45, is perhaps quite surprising, and its further increase, if possible, could raise *T*_c_ substantially.

Doping holes means entering the right upper half of the (2*n*_*p*_, *n*_*d*_)-plane. While *n*_*d*_ and *n*_*p*_ increase with doping, the ratio of the respective changes (Δ*n*_*d*_/2Δ*n*_*p*_) appears to be a family property, that is, parent materials with low *n*_*p*_ (for example, La_2_CuO_4_) add more holes to O than those with high *n*_*p*_. With electron doping we enter the lower left half of the (2*n*_*p*_, *n*_*d*_)-plane. Here, predominantly Cu holes disappear while the (large) O hole content changes only slightly. It is not apparent from the phase diagram why most parent materials can only be doped with one type of carrier. As a function of doping, *T*_c_ increases with a slope that depends on the position on the parent line, as well, and hole doping seems to be more effective in raising *T*_c_. Another important observation concerns optimal doping, that is, the doping level for which one finds the highest *T*_c_ for a given family. According to our analysis it is related to *x*=*n*_*d*_+2*n*_*p*_−1 and not particular values of *n*_*d*_ and *n*_*p*_. However, we do observe a slight increase of the optimal *x* with increasing *n*_*p*_ (decreasing *n*_*d*_). Note that the doping level *x* follows from our analysis in terms of *n*_*d*_ and *n*_*p*_ inserted into (3) and is not deduced from material chemistry. Our analysis agrees with expectations also for materials doped by interstitial O_*δ*_ where doping level *x* is often derived from the *T*_c_ dome^19^. Interestingly, the latter materials we find located in the same group, despite significant structural differences between Hg-, Tl- and Bi-based cuprates. Also the number of close CuO_2_ layers in multi-layer systems does not result in significant differences in the charge distribution.

## Discussion

In Fig. 2 we plotted only *T*_c_ in the (2*n*_*p*_,*n*_*d*_)-phase diagram, but it might be of great interest to investigate whether other cuprate properties are better presented as a function of the local charge distribution, instead of the average doping level. While further analysis is beyond the scope of our paper, we shortly discuss some other cuprate properties with regard to our phase diagram.

The Néel temperature depends on the interlayer coupling and therefore is not expected to be dominated by the charge distribution in the CuO_2_ plane. For example, YBa_2_Cu_3_O_6_ has a higher *T*_*N*_ than Pr_2_CuO_4_ and La_2_CuO_4_. It would be interesting, however, to find out how the exchange coupling (*J*) changes along the parent line. Recently, there have been contradicting reports regarding *J* in the cuprates^57^,^58^,^59^. Mallet *et al*.^58^ found no correlation between *J* and *T*_*c*,max_ in R(Ba, Sr)_2_Cu_3_O_*y*_, while Wulferding *et al*.^57^ claimed that *J* is correlated with *T*_*c*,max_ in (Ca_*x*_La_1−*x*_)(Ba_1.75−*x*_La_0.25+*x*_)Cu_3_O_6+*y*_, which was later questioned by Tallon^59^.

Structural parameters of the CuO_2_ plane such as distances, buckling, or disorder appear to show no clear trend with respect to *n*_*d*_ and *n*_*p*_. However, the apical oxygen distance from the CuO_2_ plane increases as one follows the parent line beginning from low *n*_*p*_, similar to the maximum possible *T*_c_. This behaviour and the concomitant change in density of states of Cu 4*s* was noted before^60^.

Pressure applied to underdoped cuprates usually increases *T*_c_, while the structural changes to even hydrostatic pressure can be complicated^61^. For example, specifically strained HgBa_2_CuO_4+*δ*_ can have almost identical CuO_6_ octahedra as La_2−*x*_Sr_*x*_CuO_4_, however, the large difference in their *T*_c_ values remains^62^. This might be related to the different values of *n*_*d*_ and *n*_*p*_ for these families. As a result of recent progress in anvil cell NMR^63^,^64^, it is now possible to study cuprates at high pressures also with NMR^65^, and it was found that the Cu splitting increases with pressure indicating changes in the planar hole contents^66^. However, single-crystal studies are necessary and ongoing efforts by our group aim at providing a quantitative measure of the local charge distribution as a function of pressure.

Another important issue concerns the heterogeneity of the cuprates. We know from NMR that the static charge and spin density can vary drastically within the CuO_2_ plane, in particular between different cuprate families^67^. For example, the charge density in terms of the total doping *x* may easily vary by Δ*x*≈0.05 (refs 32, 68, 69). Since *T*_c_ is not in a simple relation to this static inhomogeneity, only the average *n*_*d*_ and *n*_*p*_ appear to matter. From this, one would conclude that inhomogeneity is either not important for the maximum *T*_c_, or it is ubiquitous and dynamically averaged for NMR, depending on the chemical environment.

To conclude, NMR measures the charge distribution in the bonding orbitals in the CuO_2_ plane quantitatively, and since it reproduces the Uemura plot, that is, it finds the same ordering of families with respect to their maximum *T*_c_, we now have material chemistry parameters that are responsible for setting the highest *T*_c_ and superfluid density. These findings inspired a different perspective on the cuprate phase diagram and it is likely that the complex cuprate properties might be better understood when discussed in the context of the charge distribution in the CuO_2_ plane.

## Additional information

**How to cite this article:** Rybicki, D. *et al*. Perspective on the phase diagram of cuprate high-temperature superconductors. *Nat. Commun.* 7:11413 doi: 10.1038/ncomms11413 (2016).

## Supplementary Material

Supplementary Information Supplementary Figure 1, Supplementary Tables 1-2 and Supplementary References

## Figures and Tables

**Table 1:** List of abbreviations.

Abbreviation	Formula
Bi-2212	Bi_2_Sr_2_CaCu_2_O_8+*δ*_
CLBLCO_x	(Ca_*x*_La_1−*x*_)(Ba_1.75−*x*_La_0.25+*x*_)Cu_3_O_6+*y*_
Hg-1201	HgBa_2_CuO_4+*δ*_
Hg,Tl-1223	Hg_0.5_Tl_0.5_Ba_2_(Ca_1−*x*_Sr_*x*_)_2_Cu_3_O_8+*δ*_
La-214	La_2−*x*_Sr_*x*_CuO_4_
Nd-214	Nd_2−*x*_Ce_*x*_CuO_4_
Pr-214	Pr_2−*x*_Ce_*x*_CuO_4_
Tl-2201	Tl_2_Ba_2_CuO_*y*_
Tl-2212	Tl_2_Ba_2_CaCu_2_O_8−*δ*_
Tl-2223	Tl_2_Ba_2_Ca_2_Cu_3_O_10−*δ*_
Y-123	YBa_2_Cu_3_O_6+*y*_
Y-124	YBa_2_Cu_4_O_8_
