# Supplementary material for: Perspective on the phase diagram of cuprate high-temperature superconductors
Source: Nat Commun. 2016 May 6;7:11413. doi: 10.1038/ncomms11413 (PMC4859060; doi:10.1038/ncomms11413)
Supplement: Supplementary Information — Supplementary Figure 1, Supplementary Tables 1-2 and Supplementary References [file ncomms11413-s1.pdf]

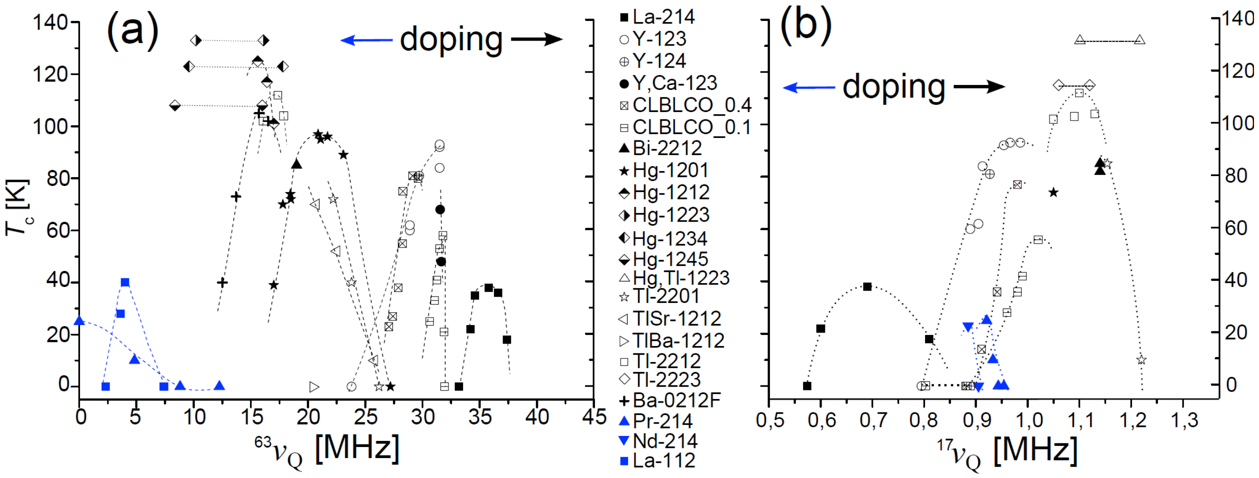

Supplementary Figure 1: **Phase diagrams of cuprates based on NMR quadrupole splittings**. Critical temperature of superconductivity T_c as a function of the NMR quadrupole splitting of the planar copper $^{63}\nu_Q$ (panel a) and oxygen $^{17}\nu_Q$ (panel b). Black and blue symbols and arrows (indicating increase of doping x) are for hole and electron doped families, respectively. Dotted lines are guides to the eye and connect different doping levels for one family. For compounds with three or more CuO_2 layers there are two different planar Cu and O sites, which have different quadrupole splittings, hence the horizontally connected data points belong to the same sample. In the case of the CLBLCO there are $^{63}\nu_Q$ data for more families [1]. Since all show the same behaviour we show only two families, for which both $^{63}\nu_Q$ and $^{17}\nu_Q$ are available. There are also more $^{63}\nu_Q$ results for cuprates containing Bi, e.g. for single layer Bi-2201 family [2]. However, the ^{63}Cu NQR spectra are very broad with at least four peaks (ranging from 27 MHz to 35 MHz), despite the fact that all Cu sites are equivalent in the ideal Bi-2201 structure without the modulation in the Bi-O layer. Very broad ^{63}Cu NQR spectra are a general feature of all Bi containing cuprates with this type of modulation [3], and we only quote one $^{63}\nu_Q$ obtained from NMR on double layer Bi-2212 single crystals [4], for which also $^{17}\nu_Q$ is available.

Family	T_c [K]	$^{63}\nu_Q$ [MHz]
La-214 ref.[5]	0	33.2
	22	34.2
	35	34.6
	38	35.8
	36	36.6
	18	37.4
Y-123 ref.[5]	0	23.8
	60	28.9
	62	28.9
	84	31.5
	92	31.5
	93	31.5
Y-124 ref.[5]	81	29.72
Y,Ca-123 ref.[11]	68 (ovd)	31.55
	48 (ovd)	31.65
Hg-1201	39 (und) ref.[13]	17.0
	70 (und) ref.[13]	17.8
	72 (und) ref.[13]	18.5
	74 (und) ref.[15]	18.46
	95 (opt) ref.[13]	21.1
	96 (opt) ref.[13]	21.7
	97 (opt) ref.[16]	20.88
	89 (ovd) ref.[13]	23.1
	0 (ovd) ref.[13]	27.2
Hg-1212	125 (opt) ref.[17]	15.6
	117 (ovd) ref.[17]	16.4
	101 (ovd) ref.[18]	17.0
	101 (ovd) ref.[20]	17.6
Hg-1223 Cu(1)	115 (und) ref.[21]	9.7
Cu(2)		13.7
Cu(1)	133 (opt) ref.[22]	10.2
Cu(2)		16.1
Hg,Cu-1223 Cu(1)	134 (opt) ref.[23]	15.3
Cu(2)		16.6
Hg-1234 Cu(1)	85 (und) ref.[25]	9.3
Cu(2)		14.8
Cu(1)	123 (opt) ref.[25]	9.6
Cu(2)		17.8
Hg-1245 Cu(1)	108 (opt) Ref.[27]	8.37
Cu(2)		16

Family	T_c [K]	$^{63}\nu_Q$ [MHz]
Bi-2212	86 (opt) ref.[4]	19.0
Tl-2201 ref.[6]	72 (ovd)	22.2
	40 (ovd)	23.8
	0 (ovd	26.2
TlSr-1212 ref.[7]	70 (ovd)	20.7
	52 (ovd)	22.5
	10 (ovd)	25.8
TlBa-1212 ref.[8]	0	20.44
Tl-2212 ref.[9]	102 (und)	16.08
	112 (opt)	17.35
	104 (ovd)	17.87
Tl-2223 Cu(1)	125 (opt) ref.[10]	11.7
Cu(2)		16.4
Cu(1)	115 (ovd?) ref.[12]	10.8
Cu(2)		17.4
Ba-0212F ref.[14]	40 (und)	12.5
	73 (und)	13.7
	105 (opt)	15.7
	102 (ovd)	16.5
CLBLCO_0.1 ref.[1]	25 (und)	30.6
	41 (und)	31.2
	58 (opt)	31.8
	21 (ovd)	31.87
	0 (ovd)	31.93
CLBLCO_0.4 ref.[1]	23 (und)	27.1
	38 (und)	27.9
	75 (und)	28.3
	81 (opt)	29.1
Pr-214 ref.[19]	0	12.2
	0	8.8
	10 (und)	4.8
	25 (opt)	0
Nd-214 ref.[19]	0	14
	25 (opt)	1
La -112	0 ref.[24]	7.4
	28 (und) ref.[24]	3.6
	40 (opt) ref.[26]	$\leq$4
	0 (ovd) ref.[24]	2.3

Supplementary Table 1: **^{63}Cu quadrupole splitting ($^{63}\nu_Q$) in the cuprates.** $^{63}\nu_Q$ for various families of cuprates in order of increasing doping ("und." - underdoped, "opt." - close to optimal doping, "ovd." - overdoped). For Tl- and Hg- families with three or more CuO_2 layers there are two Cu sites, the inner layer with Cu(1) and the outer layer Cu(2).

Family	T_c [K]	$^{17}\nu_Q$ [MHz]
La-214 ref.[5]	0	0.574
	22	0.6
	38 (opt)	0.69
	18	0.81
Y-123 ref.[5]	0	0.795
	60	0.889
	62	0.905
	84	0.913
	92	0.954
	93 (opt)	0.986
	93 (opt)	0.966
Y-124 ref.[5]	81	0.927
Hg-1201 ref.[28]	74 (und)	1.050
Tl-2201 ref.[29, 30]	85 (opt)	1.154
	10 (ovd)	1.220
Bi-2212 ref.[31, 32]	86 (opt)	1.14
	82 (ovd)	1.14
TlSr-2212	102 (und) ref.[9]	1.05
	112 (opt) ref.[9]	1.10
	104 (ovd) ref.[9]	1.13
	103 (ovd) ref.[33]	1.09
Tl-2223 O(1)	115 (ovd?) ref.[12]	1.06
O(2)		1.12
Hg,Tl-1223 O(1)	132 (opt) ref.[34]	1.101
O(2)		1.217
CLBLCO_0.1 ref.[35]	0	0.8
	0	0.89
	28	0.96
	36	0.98
	42	0.99
	55 (opt)	1.02
CLBLCO_0.4 ref.[35]	0	0.88
	14	0.91
	36	0.94
	77 (opt)	0.98
Pr-214 ref.[19]	0	0.950
	0	0.943
	10	0.933
	25 (opt)	0.921
Nd-214 ref.[19]	0	0.905
	23 (opt)	0.885

Supplementary Table 2: **^{17}O quadrupole splitting ($^{17}\nu_Q$) of planar oxygen in the cuprates.** $^{17}\nu_Q$ for various families of cuprates in order of increasing doping ("und." - underdoped, "opt." - close to optimal doping, "ovd." - overdoped). For Tl- and Hg-families with three or more CuO_2 layers there are two planar O sites, the inner layer with O(1) and the outer layer O(2).

Supplementary References

1. Keren, A., Kanigel, A. & Bazalitsky, G. Evidence for two fluids in cuprate superconductors from a nuclear resonance study of $(Ca_xLa_{1-x})(Ba_{1.75-x}La_{0.25+x})Cu_3O_y$. *Phys. Rev. B* **74**, 172506 (2006).

2. Masaki, K., Kazuyoshi, Y. & Koji, K. Evidence for antiferromagnetic order in $Bi_2Sr_2CuO_6$ phase with stoichiometric cation composition. *J. Solid State Chem*. **133**, 372-378 (1997).

3. Lipinski, I., Zalessky, A., Levanyuk, A., Mironova, G., Smirnovskaya, E. & Bush, A. ^{63}Cu and ^{63}Cu NQR lineshape in Bi-Sr-Ca-Cu-O high-T_c superconductors. *Physica C* **168**, 291-296 (1990).

4. Ishida, K., Kitaoka, Y., Asayama, K., Kadowaki, K. & Mochiku, T. Cu NMR Study in single crystal $Bi_2Sr_2CaCu_2O_8$ observation of gapless superconductivity. *J. Phys. Soc. Jpn.* **63**, 1104-1113 (1994).

5. Haase, J., Sushkov, O. P., Horsch, P. & Williams, G. Planar Cu and O hole densities in high-T_c cuprates determined with NMR. *Phys. Rev. B* **69**, 0945041 (2004).

6. Fujiwara, K. *et al.* NMR and NQR studies of superconductivity in heavily doped $Tl_2Ba_2CuO_{6+y}$ with a single CuO_2 plane. *Physica C* **184**, 207-219 (1991).

7. Magishi, K. *et al.* Magnetic excitation and superconductivity in overdoped $TlSr_2CaCu_2O_{7-\delta}$: a ^{63}Cu NMR study. *Phys. Rev. B* **54**, 10131-10142 (1996).

8. Goto, T., Nakajima, S., Kikuchi, M., Syono, Y. & Fukase, T. $^{63/65}$Cu and $^{203/205}$Tl NMR study on the antiferromagnetic phase of the Tl-based high-T_c oxide $TlBa_2YCu_2O_7$. *Phys. Rev. B* **54**, 3562-3570 (1996).

9. Gerashenko, A. *et al.* The ^{63}Cu and ^{17}O NMR studies of spin susceptibility in differently doped $Tl_2Ba_2CaCu_2O_{8-\delta}$ compounds. *Physica C* **328**, 163-176 (1999).

10. Han, Z., Dupree, R., Liu, R. & Edwards, P. ^{63}Cu NMR shift and relaxation behavior in $Tl_2Ba_2Ca_2Cu_3O_{10-\delta}$ (T_c=125 K). *Physica C* **226**, 106-112 (1994).

11. Williams, G. V. M., Krämer, S. & Mehring, M. Nuclear-quadrupole-resonance study of overdoped $Y_{1-x}Ca_xBa_2Cu_3O_7$. *Phys. Rev. B* **63**, 104514 (2001).

12. Zheng, G., Kitaoka, Y., Asayama, K., Hamada, K., Yamauchi, H. & Tanaka, S. NMR study of local hole distribution, spin fluctuation and superconductivity in $Tl_2Ba_2Ca_2Cu_3O_{10}$. *Physica C* **260**, 197-210 (1996).

13. Gippius, A. A., Antipov, E. V., Hoffmann, W. & Luders, K. Nuclear quadrupole interactions and charge localization in $HgBa_2CuO_{4+\delta}$ with different oxygen content. *Physica C* **276**, 57-64 (1997).

14. Shimizu, S. *et al.* Planar CuO_2 hole density in high-T_c cuprates determined by NMR Knight shift: ^{63}Cu NMR on bilayered $Ba_2CaCu_2O_4(F,O)_2$ and three-layered $Ba_2Ca_2Cu_3O_6(F,O)_2$. *Phys. Rev. B* **83**, 144523 (2011).

15. Rybicki, D. *et al.* ^{63}Cu and ^{199}Hg NMR study of $HgBa_2CuO_{4+\delta}$ single crystals. *http://arxiv.org/abs/1208.4690* (2012).

16. Rybicki, D. *et al.* Spatial inhomogeneities in single-crystal $HgBa_2CuO_{4+\delta}$ from ^{63}Cu NMR spin and quadrupole shifts. *J. Supercond. Nov. Magn.* **22**, 179-183 (2009).

17. Ohsugi, S., Tsuchiya, T., Koyama, T. & Fueki, K. Gapless superconductivity in overdoped Hg system; Cu-NQR study. *J. Low Temp. Phys.* **105**, 419-423 (1996).

18. Horvatić, M. *et al.* NMR investigation of $HgBa_2CaCu_2O_{6+\delta}$. *Physica C* **235**, 1669-1670 (1994).

19. Jurkutat, M., Rybicki, D., Sushkov, O. P., Williams, G. V. M., Erb, A. & Haase, J. Distribution of electrons and holes in cuprate superconductors as determined from ^{17}O and ^{63}Cu nuclear magnetic resonance. *Phys. Rev. B* **90**, 140504 (2014).

20. Julien, M.-H. *et al.* ^{63}Cu and ^{199}Hg NMR in overdoped $HgBa_2CaCu_2O_{6+\delta}$. *Physica C* **268**, 197-204 (1996).

21. Julien, M.-H., Horvatić, M., Berthier, C. & Segransan, P. ^{63}Cu NMR in the normal state of $HgBa_2Ca_2Cu_3O_{8+\delta}$. *J. Low Temp. Phys*. **105**, 371-376 (1996).

22. Magishi, K. *et al.* ^{63}Cu NMR probe of superconducting properties in $HgBa_2Ca_2Cu_3O_{8+\delta}$: a possible reason for T_c=133 K. *Phys. Rev. B* **53**, R8906-R8909 (14 1996).

23. Breitzke, H., Eremin, I., Manske, D., Antipov, E. & Luders, K. Formation of magnetic moments in the cuprate superconductor $Hg_{0.8}Cu_{0.2}Ba_2Ca_2Cu_3O_{8+\delta}$ below T_c seen by NQR. *Physica C* **406**, 27-36 (2004).

24. Mikhalev, K. *et al.* ^{63}Cu NMR study of infinite-layer compound $Sr_{1-x}La_xCuO_2$. *Physica C* **304**, 165-171 (1998).

25. Itohara, K. *et al.* Number of CuO_2 layers dependence of magnetic quantum criticality in homogeneously doped high-T_c copper oxides: A ^{63}Cu-NMR study on four-layered high- compounds $HgBa_2Ca_3Cu_4O_{8+y}$. *Physica C* **470**, S140-S141 (2010).

26. Imai, T., Slichter, C., Cobb, J. & Markert, J. Superconductivity and spin fluctuations in the electron-

doped infinitely-layered high T_c superconductor $Sr_{0.9}La_{0.1}CuO_2$ (T_c = 42 K). *J. Phys. Chem. Solids* **56**, 1921-1925 (1995).

27. Kotegawa, H. *et al.* Coexistence of superconductivity and antiferromagnetism in multilayered high-T_c superconductor $HgBa_2Ca_4Cu_5O_y$: Cu-NMR study. *Phys. Rev. B* **69**, 014501 (2004).

28. Mounce, A. M. *et al.* Absence of static loop-current magnetism at the apical oxygen site in $HgBa_2CuO_{4+\delta}$ from NMR. *Phys. Rev. Lett.* **111**, 187003 (2013).

29. Kambe, S., Yasuoka, H., Hayashi, A. & Ueda, Y. NMR study of the spin dynamics in $Tl_2Ba_2CuO_y$ (T_c=85 K). *Phys. Rev. B* **47**, 2825-2834 (1993).

30. Zheng, G., Kitaoka, Y., Ishida, K. & Asayama, K. Local hole distribution in the CuO_2 plane of high-T_c Cu-oxides studied by Cu and oxygen NQR/NMR. *J. Phys. Soc. Jpn.* **64**, 2524-2532 (1995).

31. Takigawa, M. & Mitzi, D. B. NMR studies of spin excitations in superconducting $Bi_2Sr_2CaCu_2O_{8+\delta}$ single crystals. *Phys. Rev. Lett.* **73**, 1287-1290 (1994).

32. Crocker, J. et al. NMR studies of pseudogap and electronic inhomogeneity in $Bi_2Sr_2CaCu_2O_{8+\delta}$. *Phys. Rev. B* **84**, 224502 (2011).

33. Trokiner, A. *et al.* ^{17}O NMR in high-T_c superconductor $Tl_2Ba_2CaCu_2O_y$. *Physica C* **255**, 204-210 (1995).

34. Lim, K., Lee, H. & Hur, N. An ^{17}O NMR study of $Hg_{0.5}Tl_{0.5}Ba_2(Ca_{1-x}Sr_x)_2Cu_3O_{8+\delta}$. *Physica C* **232**, 215-221 (1994).

35. Amit, E. & Keren, A. Critical-doping universality for cuprate superconductors: oxygen nuclear-

magnetic-resonance investigation of $(Ca_xLa_{1-x})(Ba_{1.75-x}La_{0.25+x})Cu_3O_y$. *Phys. Rev. B* **82**, 172509 (2010).
